# Bibliography

**Published:** 1890-05

**Authors:** 


### BIBLIOGRAPHY.

Man and His World. By John Darby. Philadelphia: J. B. Lippincott Company, 1889.

The pseudonyme of "John Darby" belongs, as is well known, to the distinguished philosopher and surgeon, Professor James E. Garretson, A.M., M.D., of Philadelphia. Amidst the exactions of a busy city physician's life, and the demands upon his time as a teacher in a leading school, he has yet found leisure to give to the world, by means of his various works, many thoughts of a character so profound and comprehensive as is seldom met with in this age.

The first part of the book, "Two Thousand Years After," was published some years since; but with the addition now of a second part, "The Philosophy of the Eternal Now," the author has given the volume the more suggestive title of "Man and his World." The groundwork of the book is the pleasing conceit that Socrates and his friends resumed, two thousand years after his tragical end at Athens, the fascinating conversation as reported by Plato; but now with its scope enlarged by reason of an acquaintanceship with modern Positivism. All those who have a mind above the merely practical affairs of life, whose thoughts love to soar into the realms of the recondite, and to grapple with what is hidden from the material eye, who find the mysteries of matter, man, mind, and soul ever interesting and enticing subjects for reflection, will discover much in this little volume that will give them pleasure and mayhap enlightenment.

Philadelphia and Its Environs. J. B. Lippincott Company, Philadelphia, 1889, pp. 232.

This little book is prepared with the same neatness and elegance that marks all the publications of this firm. Every place of interest is mentioned, copious illustrations are given, along with a good map of the city, all making a volume of so much value that visitors here and even residents can hardly afford to be without it.

Three Introductory Lectures on the Science of Thought. By F. Max Müller. Chicago: Open Court Publishing Company, 1888.

These lectures were delivered at the Royal Institution, London, and first published by the Open Court Company. The illustrious name of the author is a sufficient guarantee as to the quality of the matter, and the name of the publishing company as to the mechanical character of the book.

